# Structural and Functional Insights into Methuselah Genes of *Plutella xylostella (Lepidoptera: Plutellidae)*: Evolutionary Adaptations and Their Responses to Chlorantraniliprole

**DOI:** 10.3390/insects16111092

**Published:** 2025-10-24

**Authors:** Maryam Zolfaghari, Fei Yin, Samina Shabbir, Qichun Chen, Yong Xiao, Zhengke Peng, Zhen-Yu Li, Myron P. Zalucki

**Affiliations:** 1 Institute of Plant Protection, Guangdong Academy of Agricultural Sciences, Guangdong Provincial Key Laboratory of High Technology for Plant Protection, Key Laboratory of Green Prevention and Control on Fruits and Vegetables in South China Ministry of Agriculture and Rural Affairs, Guangzhou 510640, China; m.zolfaghari_89@yahoo.com (M.Z.); feier0808@163.com (F.Y.); chenqichun12@163.com (Q.C.); xiaoyong@gdaas.cn (Y.X.); zkpeng0827@163.com (Z.P.); 2 Guangzhou Molan Agricultural Technology Co., Ltd., Guangzhou 510000, China; 3 Department of Chemistry, The Women University Multan, Multan 60000, Pakistan; samina.shabbir@wum.edu.pk; 4 School of Biological Sciences, The University of Queensland, Brisbane 4072, Australia; m.zalucki@uq.edu.au; 5 Shandong Engineering Research Center for Enviroment Friendly Agricultural Pest Management, College of Plant Health and Medicine, Qingdao Agricultural University, Qingdao 266109, China

**Keywords:** GPCRs (*Mth*), genome-wide, phylogenetic analysis, chlorantraniliprole, RNAi, transgenic *Drosophila*

## Abstract

G protein-coupled receptors (GPCRs) are considered the largest and most variable family of transmembrane receptors regulating physiological processes such as toxicological responses and insecticide resistance development. The present study investigated the responses of Methuselah (*Mth*), belonging to GPCR family B in the Diamondback Moth (DBM), *Plutella xylostella*, to chlorantraniliprole (CAP). Genome-wide identification and phylogenetic analysis of *Pxmth* genes revealed their evolutionary relationships and functional classifications. Expression profiling demonstrated significant overexpression of *Pxmth2* in the CAP-resistant strain. Additionally, the tertiary and secondary structures of *Pxmth2* were characterized, providing insights into its functional role. Silencing *Pxmth2* via RNA interference (RNAi) reduced resistance of DBM to CAP and suppressed downstream stress-associated genes (*CYP6B6*, *CYP6B7*, *CYP6BF1*), increasing susceptibility to the insecticide. The function of *Pxmth2* was further explored using a transgenic line of *Drosophila melanogaster* engineered to overexpress the gene; flies overexpressing *Pxmth2* exhibited a significantly increased resistance to CAP compared to controls. These findings indicate that *Pxmth2* contributes to CAP resistance in DBM and highlights potential molecular targets for improving pest management strategies.

## Simple Summary

In this study, the role of Methuselah (*Mth*) genes, a G protein coupled receptor (GPCR) subfamily, in the diamondback moth (*Plutella xylostella*) in insecticide chlorantraniliprole (CAP) resistance was investigated. Genome-wide profiling and phylogenetic classification discovered eight *Pxmth* genes, of which *Pxmth2* was overexpressed in CAP-resistant strains. Structural modeling confirmed the typical GPCR characteristics of *Pxmth2*. Functional assays showed that *Pxmth2* silencing reduced CAP resistance and suppressed detoxification genes, while overexpression of the gene in transgenic *Drosophila melanogaster* increased CAP resistance. This suggests that *Pxmth2* could play a role in insecticide resistance and could be a candidate for future pest control.

## 1. Introduction

As a superfamily of membrane-bound proteins, G protein-coupled receptors (GPCRs) are distinguished by their seven-transmembrane (7TM) helical structure. They are among the most targeted molecules in pharmacology because of their various and significant biological roles; about 30–50% of all marketed medications act on these receptors [1,2,3]. In insects, GPCRs affect growth, reproduction, nutrition and other physiological processes [4]. These receptors are essential for converting ambient and extracellular cues into intracellular responses. Phospholipase C, cyclic nucleotide-gated channels, protein kinase A, cyclic adenosine monophosphate, adenylyl cyclase, diacylglycerol, and inositol triphosphate are among the downstream effectors that are activated by coupling with heterotrimeric G-proteins [5,6,7,8,9].

GPCRs in insects have become appealing targets for developing new insecticides, as they mediate key biological and physiological processes such as feeding behavior, development, and stress response that are essential for survival and adaptability [4], including insecticide resistance. Genes linked to GPCRs are overexpressed in insecticide resistant insect species [10,11]. The study of novel molecular targets, such as GPCRs, is essential for efficient pest control techniques as resistance to traditional pesticides increases [12].

The Methuselah (*Mth*) GPCR subfamily, a distinct group within the secretin-like GPCR family (family B) was initially identified in *D. melanogaster*. *Mth* genes are associated with extended lifespan and enhanced resistance to heat, oxidative stress, and starvation in hypomorphic mutants despite null mutants exhibiting pre-adult lethality highlighting a crucial contribution to development [13,14,15]. Longevity and stress tolerance imparted by *Mth* is mainly mediated via the rapamycin signaling pathway [16]. The Toll and immunodeficiency signaling pathways may also be directly involved in the functioning of *Mth*-like gene (*Tcmthl1*) with respect to stress and lifespan in *Tribolium castaneum* [17]. In *Dastarcus helophoroides*, three *Mth*-like genes were differentially transcribed in a variety of tissues and at different phases of development, implying that these genes participate in development. Further, their expression in adults was induced by heat, oxidative stress, starvation, and aging, suggesting that they play an important role in aging, reproduction and defense mechanisms against environmental stressors [18].

The diamondback moth (DBM), *Plutella xylostella* (Lepidoptera: Plutellidae), is a major global pest causing substantial annual economic losses in cabbage fields [19]. Its capacity to develop resistance to all pesticide classes has made management increasingly challenging [20]. Although several studies have investigated GPCRs in *P*. *xylostella* [21,22,23], a limited body of research has addressed the role of the *Mth* subfamily in stress regulation in this pest, despite its recognized importance in other insects.

Chlorantraniliprole (CAP) is an anthranilic diamide insecticide that targets the ryanodine receptor (RyR) in insect muscle cells and is widely used against lepidopteran pests, including DBM. However, CAP resistance has been reported in DBM populations from several countries [24], mainly due to enhanced detoxification through transport proteins and enzymes such as CYP450s, CarEs, and GSTs. Despite cases of resistance, CAP remains effective, highlighting the need for strategies to delay resistance increasing and preserve its long-term efficacy [25].

Accordingly, the present study aims to evaluate the evolutionary relationships and structural characteristics of potential *Mth* gene candidates using bioinformatic methods and phylogenetic analysis. The secondary and tertiary structures of one candidate, *Pxmth2*, was predicted, the gene’s response to CAP exposure assessed, and its developmental expression pattern analyzed. Moreover, the potential role of *Pxmth2* in lifespan regulation and pesticide resistance is examined using functional genomics and reverse genetics techniques. It is expected that these findings will enhance our understanding of *Mth* genes in insects and offer novel strategies for addressing the serious pest issues posed by *P*. *xylostella*.

The Methuselah genes *PxMth2* plays a central role in regulating stress tolerance and CAP resistance in *P. xylostella*, with its expression being developmentally regulated and by responding to pesticide exposure. Altering *PxMth2* expression is expected to affect downstream detoxification pathways, thereby influencing the insect’s survival and adaptability.

## 2. Material and Methods

### 2.1. Insects

A pesticide-susceptible *P. xylostella* strain gathered in Guangdong, China (2002) was maintained pesticide-free in the laboratory. In 2023, a CAP-resistant strain was isolated from Huizhou (Guangdong) cabbage fields (E 113.9808°, N 23.127062°), where intensive and long-term use of CAP had imposed strong field resistance. After collection, the colony was reared on *Brassica rapa* in controlled conditions (a temperature of 25 degrees Celsius, relative humidity of 65%, and a 16 h light: 8 h dark cycle) for three generations without additional insecticide exposure to ensure stabilization. The larvae were fed *B. rapa*, while the adults were given a 10% honey solution, with no pesticide exposure.

### 2.2. Leaf-Dip Bioassays

To determine the LC_50_, a preliminary bioassay was first performed to identify doses producing approximately 10% and 90% mortality (27 and 85 mg/L, respectively). Based on these results, three intermediate concentrations were chosen using geometric (logarithmic) sequence according to [26]. yielding five test doses: 27, 34, 47, 63, and 85 mg·L^−1^. This spacing approximates a constant ratio between successive concentrations (mean ratio ≈ 1.33), as recommended for probit analysis. The toxicity of CAP was evaluated using a standard leaf-dip bioassay with cauliflower leaves [27]. Commercial formulations were serially diluted in distilled water containing 0.1% Triton X-100 (*v*/*v*). Leaf discs (5 cm diameter) were dipped into each concentration (27, 34, 47, 63, and 85 mg/L for the HZ-R strain) for 30 s, air-dried at room temperature for 1 h, and individually placed in 7 cm plastic Petri dishes. Controls consisted of leaf discs dipped in distilled water with 0.1% Triton X-100. Each concentration included three replicates of ten third-instar larvae of the same age. Mortality was recorded after 48 h; larvae were considered dead if they failed to move in a coordinated manner when touched with a brush. Dose–response data were analyzed using Probit regression on log-transformed concentrations to estimate LC_50_ values and their 95% confidence intervals. The PoloPlus software (Version 2.0) was used for model fitting. The LC_50_ for pesticide and each strain was estimated from experiments with <10% control mortality.

### 2.3. Insecticides

CAP (50 g L^−1^ SC) used in this experiment was obtained from DuPont Agricultural Chemicals Company (Ltd., Wilmington, DE, USA).

### 2.4. Documentation of Mth Genes in the P. xylostella Genome

*Mth* genes were identified by a BLASTP search against the *P. xylostella* genome (https://www.ncbi.nlm.nih.gov/datasets/genome/GCF_932276165.1/, 25 March 2025) using reference sequences from *D. melanogaster*, *Bombyx mori*, and *Musca domestica*. Candidate genes containing 7TM domains were verified by TMHMM, version 2.0 (http://www.cbs.dtu.dk/services/TMHMM/, accessed on 25 March 2025) [28]. In addition, likely subcellular localization was identified using WoLF PSORT (http://www.genscript.com/psort/wolf_psort.html, accessed on 25 March 2025) based on available data [29], and nuclear localization signals were identified through the NLSdb database (https://nls-mapper.iab.keio.ac.jp/cgi-bin/NLS_Mapper_form.cgi, accessed on 25 March 2025). Further, molecular weight, isoelectric points (pI), and GRAVY scores were computed by ExPASy (https://web.expasy.org/protparam/, accessed on 25 March 2025) (Table 1).

### 2.5. Phylogenetic Analysis and Chromosomal Mapping

First, a phylogenetic tree was constructed containing *Mth* protein sequences from *P. xylostella*, *D. melanogaster*, and *B. mori.* Then the deduced GPCR protein sequences were aligned using the default parameters in Clustal X2.0 for multiple sequence alignment. Phylogenetic trees were generated with MEGA 5.0 using the neighbor-joining technique. Further, pairwise gap elimination was executed under the bootstrap analysis of 1000 replicates as specified by [30]. The chromosomal sites of GPCRs were identified using TBtools v1.120 (https://github.com/CJ-Chen/TBtools, accessed on 1 May 2025) [31]. Finally, gene mapping of chromosomes was performed using MG2C v2.1 (http://mg2c.iask.in/mg2c%5Fv2.1/, accessed on 1 May 2025) [32].

### 2.6. Gene Structure and Motif Analysis

The Gene Structure Display Server (http://gsds.cbi.pku.edu.cn/, accessed on 1 May 2025) was used to examine the intron/exon organization of genomic DNA and *P. xylostella* complementary DNA (cDNA) sequences to investigate the structure and distribution of *Pxmth* genes [33]. A visual summary of gene structures was offered by this analysis. In addition, domains and conserved motifs within *Pxmth* candidate amino acid sequences were estimated by MEME (https://github.com/cinquin/MEME?utm_source=chatgpt.com, accessed on 1 May 2025) based on previous research [34]. MEME parameters were adjusted to allow a maximum of 10 motif repeats, with preferred motif lengths ranging from six to two hundred amino acids. The aim of this strategy was to find conserved elements that could give insight into the possible roles of the *Pxmth* genes.

### 2.7. Protein Structure Analysis

The homology models of *Pxmth2* domains were created by applying SWISS-MODEL (https://swissmodel.expasy.org, accessed on 16 May 2025) and AlphaFold, version 2. The TMHMM-2.0 server predicted membrane localization.

#### 2.7.1. Transmembrane Domain Prediction

The TM regions of *Pxmth* were predicted by the TMHMM-2 server (https://services.healthtech.dtu.dk/service.php?TMHMM-2.0, accessed on 16 May 2025). The input protein sequence was retrieved from the UniProt database and analyzed using TMHMM (https://services.healthtech.dtu.dk/service.php?TMHMM-2.0, accessed on 16 May 2025) to identify TM helices (TMHs). The results, visualized as a TM topology plot, confirmed the presence of multiple TM domains in the protein structure.

#### 2.7.2. Ramachandran Plot Analysis

To study the stereochemistry of the protein structure, a Ramachandran plot was generated using PROCHECK (https://www.ebi.ac.uk/thornton-srv/software/PROCHECK/, accessed on 20 May 2025). The protein’s three-dimensional structure, predicted or experimentally determined, was subjected to PROCHECK analysis to assess backbone dihedral angles (Φ and Ψ). The plot categorizes residues into favored, allowed, and outlier regions, helping evaluate the protein’s structural integrity. Residues in the favored regions indicated well-folded regions of the protein, while outliers were flagged for potential structural issues.

### 2.8. Multiple Sequence Alignment and Phylogenetic Analysis

The genes and amino acid sequences related to the *Pxmth2* gene were obtained from a transcriptome database and the NCBI database (http://www.ncbi.nlm.nih.gov/BLAST, accessed on 30 May 2025), respectively. Sequence alignments were performed with Clustal Omega v1.2.4 (https://www.ebi.ac.uk/Tools/msa/clustalo/, accessed on 30 May 2025), and aligned sequences were annotated with ESPript 3.0 to visualize conserved residues and secondary structure elements in the form of α-helices and β-sheets. The phylogenetic tree was developed by the maximum likelihood technique of MEGA-X with 1000 bootstrapped replicates. The resulting tree was visualized and annotated, along with bootstrap values for major nodes. Scale bars show evolutionary distance in terms of substitutions per site.

### 2.9. Signal Peptide Prediction

Signal peptides were searched with the aid of SignalP-6.0 [35], and ProP1.0 software [36] was utilized for predicting residues of potential cleavage sites within the preproteins and to perform a general preprotein convertase prediction.

### 2.10. Expression Profiling of Mth Genes Under CAP Exposure

Eight gene-specific primers were designed to quantify the expression of *Mth* genes in CAP-susceptible and -resistant *P. xylostella* strains (see Table S1 Supplementary Data), Invitrogen Trading Company, Ltd., Shanghai, China). Bioassays determined LC_30_ (47 mg/L) and LC_50_ (63 mg/L) values for CAP-resistant strains, which were applied to 100 larvae per treatment. Control groups were treated with water and TritonX-100 solutions. Samples were collected 6, 24, and 48 h post-treatment. Total RNA was extracted from pooled samples of five larvae per treatment using the EASYspin RNA isolation kit (Biomed, Beijing, China) based on the manufacturer’s instructions. First-strand cDNA synthesis was conducted by employing e M-MLV reverse transcriptase (Takara Bio Inc., Shiga, Japan). Quantitative polymerase chain reaction (qPCR) was performed in triplicate with a Rotor-Gene thermal cycler (Bio-Rad Laboratories, Hercules, CA, USA) using reactions made of 1 µL of cDNA, 10 µL of green qPCR SuperMix, 8.2 µL of ddH_2_O in a 20 µL total volume, and 0.4 µL of each primer (0.2 µM). Primer specificity was checked by melting curve analysis, confirming single-amplicon generation. The housekeeping gene ribosomal protein RPL32 (GenBank: AB180441) was utilized as a reference, and relative transcript levels were determined by the 2^^−∆∆CT^ technique.

### 2.11. Functional Verification of Pxmth2 by RNA Interference (RNAi)

Gene-specific primers targeting distinct segments of the *Pxmth2* gene (GenBank accession number XM038115253.2) were designed using genomic *P. xylostella* data and the SnapDragon double-stranded RNA (dsRNA) design tool (accessed 25 September 2023). The cDNA synthesized from DBM larval RNA was employed for the amplification of *Pxmth2* segments with forward and reverse primers encompassing the T7 polymerase promoter sequence at their 5′ ends (forward primer 5′-TAATACGACTCACTATAGGGCAGTCGAGCTTCTTCTGGCT-3′ and reverse primer 5′-TAATACGACTCACTATAGGGTAGAGGCCGTATCGTTGCTT-3′). Amplification was performed by initial denaturation at 95 °C for 3 min, 35 cycles of denaturation at 95 °C, annealing at 60 °C, and extension at 72 °C for 45 s each, and final extension at 72 °C for 5 min. The purified amplicons by Gel Extraction Mini Kit (TransGen Biotech, Beijing, China), served as templates for in vitro transcription by applying the RiboMAX T7 system (Promega, Madison, WI, USA), according to the instructions of the manufacturer. The synthesized dsRNA targeting *Pxmth2* was treated with DNase I to remove residual DNA templates, precipitated with ethanol, and dissolved in RNase-free water. Third-instar larvae were injected with 300 ng/larva dsRNA targeting the *Pxmth2* gene using a microinjector (Sarasota, FL, USA), into the hemocoel between the second and third legs on the thorax. A dsRNA targeting green fluorescent protein (GFP, GenBank accession: MN623123.1) was synthesized as a control. After dsRNA injection, larval survival was monitored at 24 h post-injection. Only larvae that survived the injection and showed normal feeding behavior were included in subsequent gene expression and bioassay analyses. Statistical analysis of silencing efficiency was conducted using three independent biological replicates, and the detailed results are provided in (Table S1, Supplementary Data). To determine transcript levels a qPCR was performed 24h after the injection. Total RNA was prepared from the TRIzol^®^ reagent (Invitrogen, Carlsbad, CA, USA), and its quantity and quality were measured by a NanoDrop™ spectrophotometer at OD260/280 ratios and agarose gel electrophoresis (Thermo Scientific, Wilmington, DE, USA). First-strand cDNA was synthesized using the cDNA Synthesis SuperMix kit (Transgen) and the TransScript One-Step gDNA Removal. qPCR reactions were conducted under the initial denaturation at 94 °C for 30 s, followed by 40 cycles of 94 °C for 5 s and 60 °C for 30 s (primers are provided in Table S1, Supplementary Data). The relative expression of genes was normalized against the reference gene ribosomal protein RPL32, and the comparative expression was quantified by the 2^^−∆∆Ct^ method. Leaf-dip bioassays were conducted 24 h post-injection to investigate the function of *Pxmth2* in CAP susceptibility with LC_50_. Mortality due to mechanical injury from injection was excluded from the statistical analysis to ensure that the observed effects were specifically due to RNAi gene silencing. Each treatment group contained three biological replicates replicate with 10 larvae per replicate (total 30 larvae per treatment), in which dsRNA targeting GFP (dsGFP) was utilized as a control. Mortality was recorded after 48 h. The mortality data were analyzed by probit analysis using PoloPlus software (LeOra Software, version 1, Parma, MO, USA) to estimate the LC_50_ value.

### 2.12. Generation of UAS-Pxmth2 Transgenic Drosophila

To determine whether *Pxmth2* regulates resistance to CAP, transgenic *D. melanogaster* strains overexpressing *Pxmth2* were generated by Fungene Biotech (Qidong, China). Overexpression was confirmed by qPCR, and functional effects were assessed through adult mortality bioassays. CAP toxicity was tested following the protocol of [37]. Briefly, CAP was serially diluted into six concentrations (1, 0.5, 0.25, 0.125, 0.062, 0.031, 0.0156 units mg/L) using 10% honey solution as the solvent. Groups of 20 female flies were placed in each vial, with three replicates per concentration. A control group (*w1118*) was treated with 10% honey solution without CAP. Mortality was recorded after 48 h, with all ataxic flies counted as dead. All flies were maintained at a temperature of 25 degrees Celsius, relative humidity of 65%, and a 12 h light: 12 h dark cycle.

### 2.13. Statistical Analysis

Mortality data from leaf-dip bioassays were analyzed using PoloPlus software to calculate LC_30_ and LC_50_ values with 95% confidence intervals based on probit analysis. For RT-qPCR experiments, relative gene expression was calculated using the 2^^−ΔΔCt^ method. Differences in gene expression between treatments or strains were evaluated using Student’s *t*-test (*p* < 0.05). Data visualization and statistical comparisons were performed using GraphPad Prism (version 9.0).

## 3. Results

### 3.1. Identification and Distribution of Mth-Encoding Genes in the P. xylostella Genome

To detect *Mth* genes in the *P. xylostella* genome, a sequence alignment was conducted using BLAST v1.2.4 (https://www.ebi.ac.uk/Tools/msa/clustalo/, accessed on 30 May 2025), against known *Mth* genes from *B. mori*, *D. melanogaster*, and *Musca domestica*. *B. mori* was selected because it is a well-studied model species in Lepidoptera with comprehensive genomic data available, while *D. melanogaster* and *M. domestica* were included due to the extensive functional characterization of *Mth* gene families in these dipteran insects. Eight potential *Mth* genes were identified in the *P*. *xylostella* genome by matching with the reference sequences. The NCBI database was then used to download available basic information about the *Mth* genes including the protein, coding, and genomic sequences, gene name, chromosome position, chromosomal number, protein length, molecular weight, GRAVY, exon/intron number, and pI value (Table 1). The calculated molecular weights of the *Mth* potential candidates were in the range of 45.86 Da (*Pxmth3*, 407aa) for the shortest to 79.82 Da (*Pxmth4*, 715aa) for the longest protein sequence. The determined pI of the *Mth* candidates ranged from 6.23 (*Pxmth1*) to 8.76 (*Pxmth7*).

### 3.2. Characterization of Pxmth in P. xylostella

**Figure 1:**
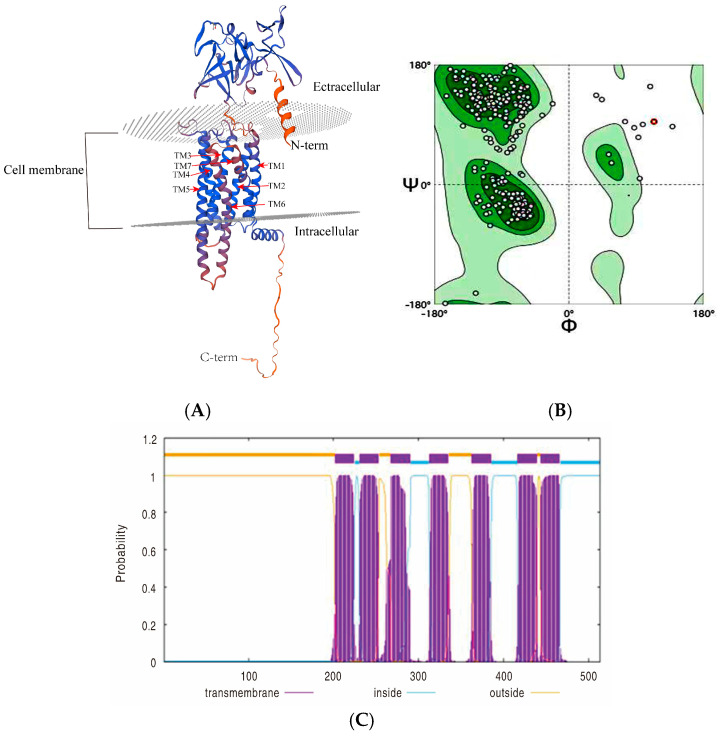
(**A**) Ribbon diagram displaying the global structure of *PxMth2* dividing the intracellular and extracellular domains, the transmembrane regions (TM1–TM4) span the cell membrane, with the N-terminal facing the extracellular space and the C-terminal located intracellularly. Figures were generated using the molecular viewing program Swiss Model. (**B**) Confirmation of *Mth2* protein in *P. xylostella* via Ramachandran plots and 3D verification via the SWISS-MODEL, Ramachandran plot showcases the allowed and favored regions for backbone dihedral angles, as well as the phi and psi bonds (Φ and Ψ) of amino acids in a protein structure. The green areas indicate highly favored conformations, while the gray dots represent individual residues, and the red dot highlights a specific outlier. (**C**) TMHMM posterior probabilities for *PxMth2* (XP_037971181.2) illustrating predicted transmembrane regions (purple), intracellular regions (blue), and extracellular regions (orange) along the protein sequence, Peaks and plateaus highlight the distinct topology across the sequence length (1–500 amino acids).

A full-length *Mth* gene (LOC119693055) was detected in *P. xylostella*, with an open reading frame of 554 bp encoding a total of 184 amino acids. The molecular mass and the pI were predicted as 57.10 kDa and 8.05, respectively. The structure of the *Pxmth2* homology model encompasses the 7TM domain, the extracellular domain (ectodomain), and the intracellular domain (Figure 1A).

**Figure 2:**
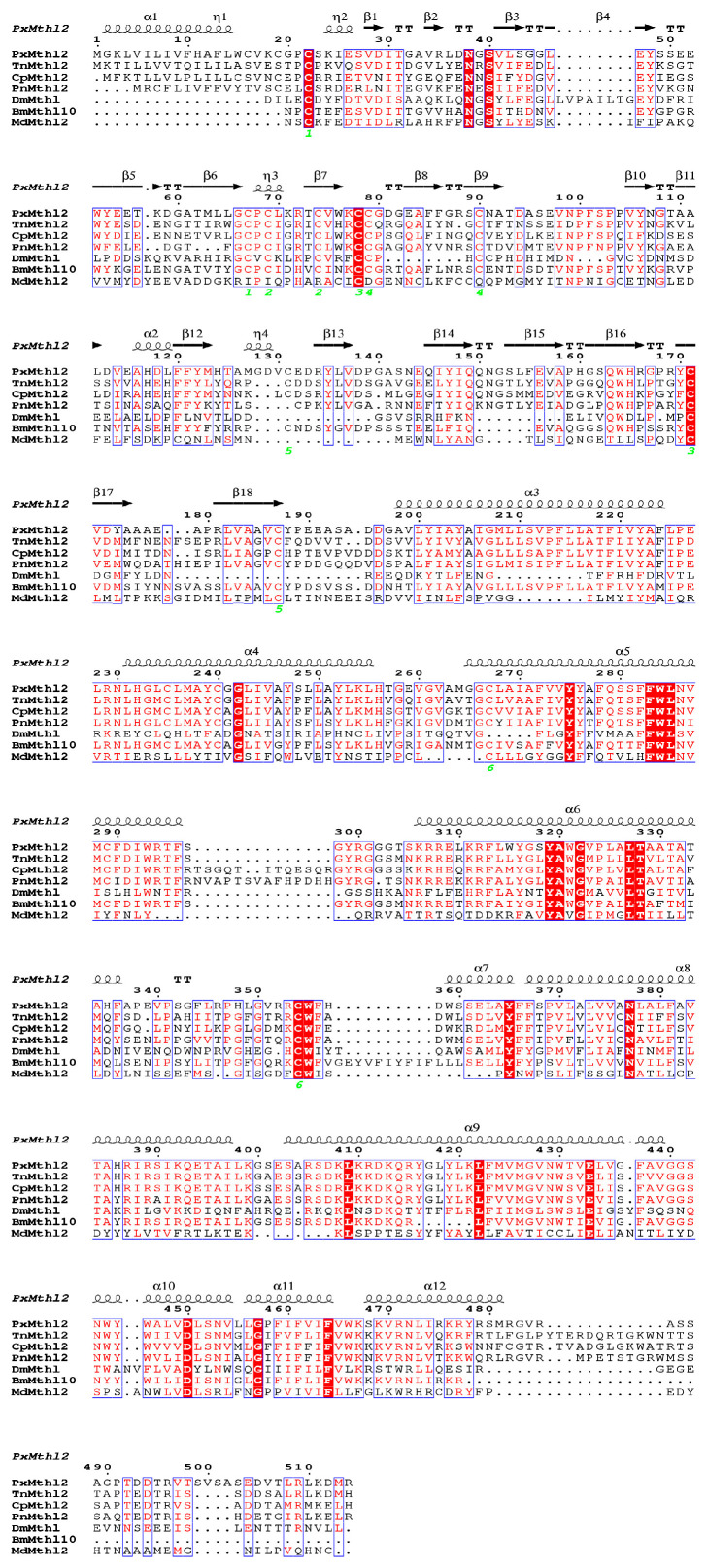
Structural and phylogenic analysis of *PxMth2* (XM_038115253.2). *P. xylostella* (*PxMth2*), *Trichoplusia ni* (*TnMth2*), *Cydia pomonella* (*CpMth2*), *Pieris napi* (*PnMth2*), *Drosophila melanogaster* (*DmMth*), *Bombix mori* (*BmMth*), and *Musca domestica* (*MdMth2*). The alpha helix, beta sheet, random coil, and beta turn are identical to α, β, η and T, respectively. Conserved residues are highlighted in red, and the intensity of shading indicates the degree of conservation.

The A0A811WXK3.1 template was employed to model the ectodomain of the *Mth* gene. Model quality for the ectodomain was reported by the I-TASSER server as a confidence score (C-score) of 0.78, and the TM score was 0.58 ± 0.50. The crystal structure template for modeling the intracellular domain and the *Mth2* gene TM domain contained class B GPCRs. *Mth*, similar to secretin receptor family members, has a large-scale N-terminal ectodomain, which probably constitutes the ligand binding site (Figure 1A). Model superposition and sequence alignment demonstrated a total of 10 conserved cysteine residues located in the ectodomain of *Mth* and *Mth*-like proteins. Conserved residues, including hydrophobic regions and charged residues, were observed in ligand-binding domains, particularly around the β-sheets and α-helices critical for receptor function. The *PxMth2* sequence exhibited unique features, including an extended hydrophobic region and slight variations in the conserved ligand-binding motifs, which could have functional implications. Structural annotations identified secondary elements, such as α1, α2, and β1, consistent across species, supporting the evolutionary conservation of the GPCR framework (Figure 2).

The TMHMM indicated that the protein PxMth2 (XP_037971181.2), which has a total length of 514 amino acids, contains seven TMHs at positions 202–224, 231–253, 268–290, 313–335, 363–385, 417–439, and 444–466. The N-terminal (1–201) and the spaces between TMHs were the main locations for regions outside the membrane, whereas positions 225–230, 291–312, 336–362, and 386–416 were the locations for regions within. In accordance with the properties of TM proteins, the general structure indicated that this protein is membrane-bound and has alternating intracellular and extracellular domains (Figure 1C).

The Ramachandran plot for the protein structure revealed that the majority of residues fall within the energetically preferred regions, which are depicted by the darkest green areas. These regions are related to the common α\alphaα-helical and β\betaβ-sheet conformations. A few residues are located in allowed regions (light green), and very few outliers were observed, including one prominently marked in red, which represents a residue with unusual dihedral angles. The distribution of angles suggests that the protein adopts a well-defined secondary structure with minimal steric clashes or deviations (Figure 1B).

### 3.3. Phylogenetic and Domain Analysis

**Figure 3:**
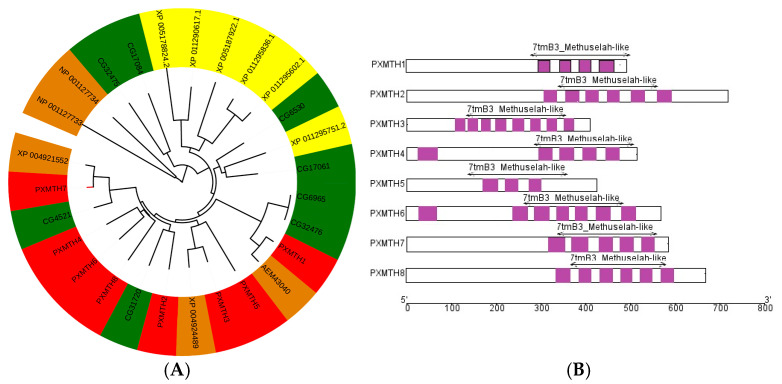
Phylogenetic tree displaying *Mths* receptors from *D. melanogaster*, *M. domestica*, *P. xylostella*, and *B. mori*, generated using the neighbor-joining method. Receptors from *P. xylostella*, *M. domestica*, *D. melanogaster*, and *B. mori* are highlighted in pink, yellow, green, and brown, respectively. The scale bar indicates p-distance (**A**). Domain analysis for *Mth* genes. The CDD database identified the conserved domain structures of 8 *Mth* genes from *P. xylostella*. Purple boxes represent the *Mths* domain (**B**).

A phylogenetic tree based on multiple sequence alignment was developed to study the evolutionary and phylogenetic connections among the proteins of *P. xylostella*, *B. mori*, and *D. melanogaster* (Figure 3A). The majority of *Mth* genes had orthologous connections with proteins in *B. mori* and *D. melanogaster*, but there was no direct linkage with *M. domestica.* Additionally, there were multiple cases of *Mth* gene deletion and duplication in *B. mori* and *D. melanogaster*. The structural analysis demonstrated that most *Mth* candidates in *P. xylostella* possess multiple TM domains, implying that *Mths* in *P. xylostella* are highly conserved (Figure 3B).

### 3.4. Gene Structure and Conserved Motif Analysis

**Figure 4:**
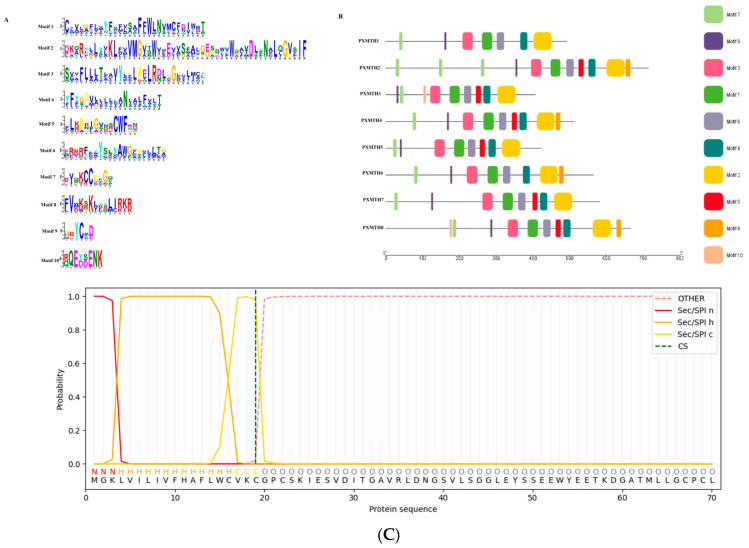
(**A**) Sequences of the ten motifs identified by MEME (https://github.com/cinquin/MEME?utm_source=chatgpt.com, accessed on 1 May 2025), (**B**) Venn diagram illustrating motif distribution across domains, with the number of conserved domains limited to a maximum of ten, and (**C**) Signal peptide 6.0 prediction for the G-protein-coupled *Mth2* isoform X2 from *P. xylostella*. The graph displays the probability of signal peptide regions: Sec/SPI n-region (red), h-region (orange), c-region (yellow), and cleavage site (CS, green dashed line). The sequence prediction highlights the presence of a signal peptide, aiding in functional localization analysis.

The MEME tool was employed to assess the motifs in the eight *Mth* proteins, and the results confirmed that there were generally 10 conserved motifs. Three highly conserved sequence motifs were found in the majority of *Mth* proteins and subsets of *Mth* proteins shared more conserved motifs. These findings show that *Mth* proteins are highly conserved (Figure 4A,B); nonetheless, this subfamily may have additional, as-yet-unidentified activities that require further research.

SignalP 6.0 predictions further validated the presence of a signal peptide within the N-terminal region of the protein. The cleavage site was predicted at position 20, as indicated by the green dashed line, and the Sec/SPI (Signal Peptide) probabilities depicted a clear transition from the n-region (red) to the h-region (yellow) and finally to the c-region (orange). This indicates that the protein undergoes typical signal peptide processing and is subsequently processed as a membrane-bound receptor. The presence of a functional signal peptide aligns with its role as a membrane protein involved in environmental sensing or ligand interaction (Figure 4C).

**Figure 5:**
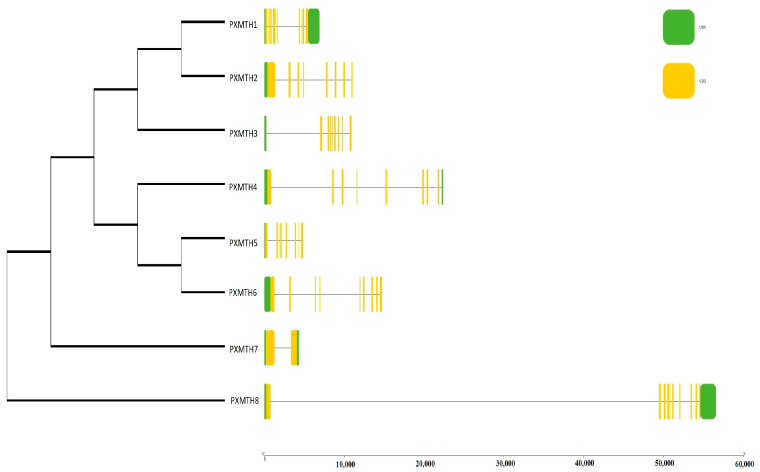
The *Mth* gene structure analysis of *P. xylostella*. Untranslated 5-3 regions (UTR), exons, and introns are displayed in the legend on the right-hand side. Green color (UTR), yellow color (Exon) and the line between Exones part is Intron.

The exon–intron structure and conserved motifs of *Mth* genes were investigated to gain insight into the evolutionary development of the *Mth* gene family within the *P. xylostella* genome. There were between 7 and 11 exons and between 8 and 12 introns (Figure 4B). With a few exceptions within the same sub-clade, the majority of *Mth* genes displayed parallel structures. *PXMH8*, *PXMTH4*, *PXMTH6*, *PXMTH2*, *PXMTH3*, *PXMTH1*, and *PXMTH7* were identified to include the longest exon–intron structures (Figure 5).

### 3.5. Expression Profiles of Mth Receptors in Response to Chlorantraniliprole

**Figure 6:**
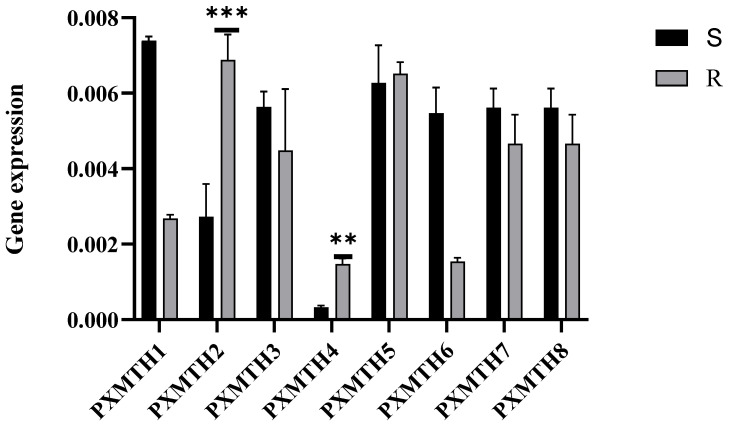
The expression levels of GPCR genes quantified by the 2^^(−∆∆Ct)^ technique. Bars indicate the relative expression levels of GPCR genes, with statistical comparisons to the control group conducted using Student’s *t*-test (** *p* < 0.01, and *** *p* < 0.001). GPCR genes with relative expression values ≥ 1 are classified as upregulated, while those with values < 1 are grouped as downregulated.

To explore the potential contribution of differentially expressed *Mth* genes to detoxification metabolism in *P. xylostella*, we examined the expression levels of these receptors before exposure to CAP using RT-qPCR in both susceptible and resistant strains (Figure 6). The results showed that *PxMth2* and *PxMth4* were upregulated in the resistant strain, with *PxMth2* exhibiting a much higher expression level compared to *PxMth4.*

**Figure 7:**
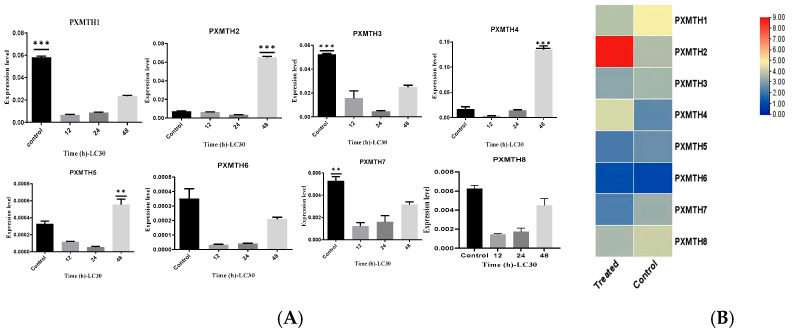

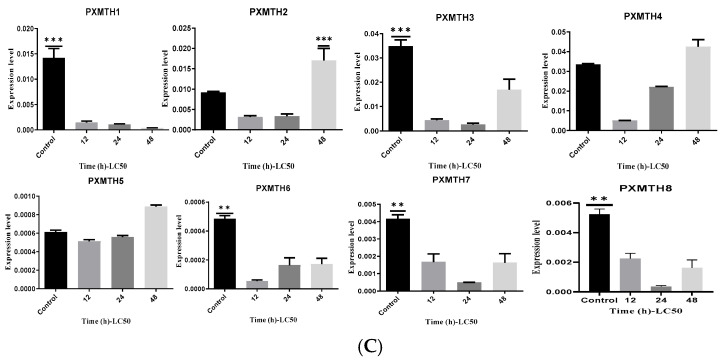
(**A**) *Mth* gene expression at LC_30_ in strains susceptible and resistant to chlorantraniliprole, (**B**) expression at the LC_50_ level, and (**C**) a heat map displaying log2-transformed FPKM values for the expression profiles of *Mth* genes across different *Mth* genes of the CAP-resistant strain. The 2^^(−∆∆Ct)^ technique was applied to quantify gene expression, and bars were employed to show relative expression. Student’s *t*-test was utilized for statistical comparisons to the control; ** *p* < 0.01, *** *p* < 0.001. High to low expression is marked by red to blue (**C**).

To further evaluate the possible role of these differentially expressed genes, concentrations of LC_30_ (47 mg/L) and LC_50_ (63 mg/L) were utilized to assess the regulation of *Mth* gene expression in *P. xylostella* (Table 2). *PXMTH2* and *PXMTH4* were significantly up-regulated after 48 h of exposure to the LC_30_ dose (Figure 7A). Similarly, after 48 h of LC_50_ treatment, *PXMTH2*, *PXMTH4*, and *PXMTH5* showed increased expression, with *PXMTH2* displaying a notably higher increase compared to the others. These findings suggest that up-regulated *Mth* gene expression may be associated with CAP detoxification metabolism.

Transcriptome analysis of the eight *Mth* genes showed differential expressions in response to CAP treatment. *PXMTH2* was the most highly expressed gene (Figure 7B).

### 3.6. Investigation of RNAi of Pxmth2 in Resistance to CAP

**Figure 8:**
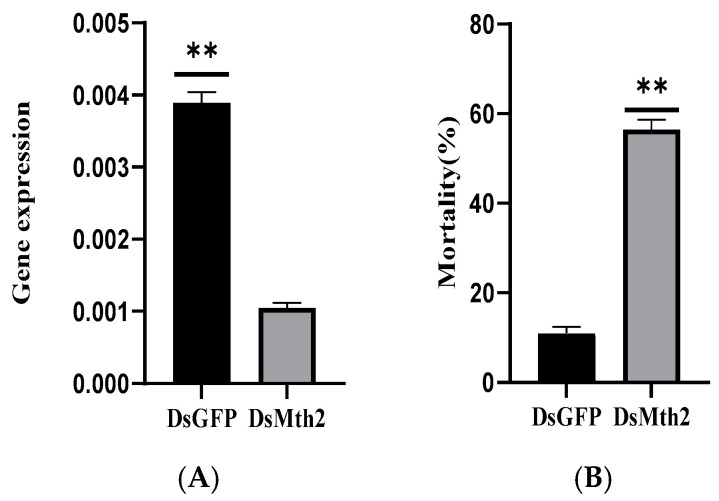
*Pxmth2* relative expression (**A**) and mortality percentage (**B**) in resistant strain larvae after *Pxmth2* RNAi; GFP: *green fluorescent protein; DsRNA:* double-stranded RNA. Significant differences were indicated by various letters above the bars, and statistical differences were evaluated using a *t*-test (*p* < 0.05). ** denotes a highly significant difference at *p* < 0.01.

For several lepidopteran species, including *P. xylostella*, RNAi has been used as a functional genomics technique [38,39,40,41,42,43,44]. In this study, RNAi was utilized to knock down genes in third-instar *P. xylostella* larvae to investigate the functions of *Pxmth2* in resistance to CAP. The mRNA levels of *Pxmth2* were much lower in dsRNA injected larvae compared to those injected with GFP RNAi 24 h after injection (Figure 8A), indicating successful *Pxmthl2* silencing.

Next, we assessed how *Pxmth2* silencing affected the mortality of *P*. *xylostella* larvae under CAP stress. After being exposed to 63 mg/L CAP for 24 h, the cumulative mortality in larvae microinjected with dsRNA for *Pxmth2* was significantly higher than that of the control group injected with dsGFP. The cumulative mortality rate of the experimental larvae was 57%, whereas the control group’s mortality rate was just 11% (Figure 8B). These findings demonstrate that third-instar *P*. *xylostella* larvae’s resilience to low levels of CAP stress was diminished by *Pxmth2* silencing.

**Figure 9:**
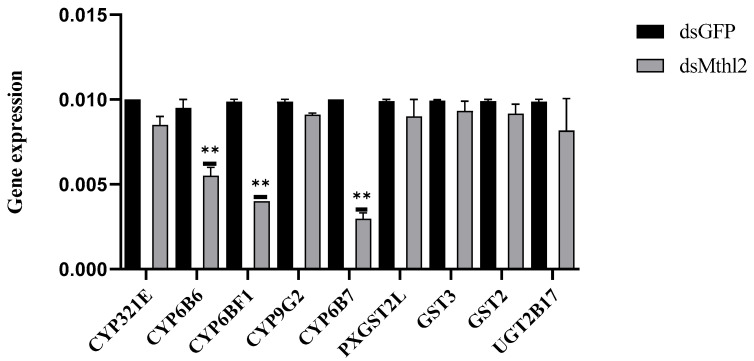
Impact of *Pxmth2* RNAi on stress resistance gene expression. Twenty-four hours after *Pxmth2* RNAi treatment, the relative mRNA levels of *P. xylostella* genes were assessed, normalized to RPL32 expression, and contrasted with levels after GFP RNAi treatment. The data are shown as means ± SE (*n* = 3). A *t*-test (*p* < 0.05) was employed to find statistical significance between the treatment and control (dsGFP) groups; significant differences were identified by various letters above the bars. ** denotes a highly significant difference at *p* < 0.01.

Longer lifespans and higher resistance to stress have been associated with *Mth* and *Mth*-like genes [15,45,46]. The expression levels of these target genes following *Pxmth2* silencing were studied to determine whether *Pxmth2* regulates stress resistance-related genes, such as *GSTs*, *P450s*, and *UGT*. Following *Pxmth2* silencing, the expression of *CYP6B6*, *CYP6BF1*, and *CYP6B7* was decreased (Figure 9), implying that these genes are probably engaged in stress resistance control.

### 3.7. Pxmth2 Overexpression Confers CAP Resistance in Drosophila

To evaluate the impact of *Pxmth2* overexpression on CAP resistance, bioassays were performed using transgenic *D. melanogaster* lines. The survival data revealed that flies overexpressing *Pxmth2* exhibited significantly reduced sensitivity to CAP compared to the wild-type (w1118) controls (Table 3). The LC_50_ for the transgenic line was 0.7 mg/L, a three-fold increase relative to the WT line (LC_50_ = 0.24 mg/L), indicating enhanced resistance. QPCR analysis was performed comparing UAS-*PxMth2* lines with the *w1118* control. The results confirmed significant overexpression of *PxMth2* in transgenic flies, while no detectable expression was observed in wild-type controls. These findings suggest that *Pxmth2* overexpression confers a substantial detoxification advantage, potentially contributing to increased tolerance to CAP.

**Figure 10:**
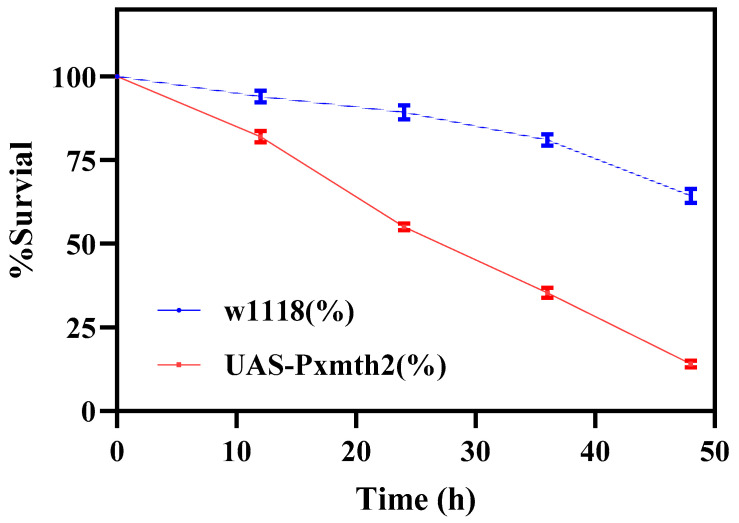
Survival rate of *D. melanogaster* lines following CAP exposure. Red line indicates control strain (w1118), and blue line indicates transgenic *UAS-Pxmth2* flies. Survival was recorded at 12 h intervals up to 48 h post-treatment.

The survival curves clearly demonstrated that overexpression of *Pxmth2* enhanced tolerance to CAP in transgenic *D. melanogaster* compared to the control strain (*w1118*) (Figure 10). After 48 h of CAP exposure, only 18% of the control flies survived, whereas 65% of the *UAS-Pxmth2* line remained alive. The survival decline was significantly slower in the transgenic flies across all time points, confirming that *Pxmth2* overexpression confers a protective effect against CAP toxicity.

## 4. Discussion

*Mth* genes in *P. xylostella* were identified and analyzed to explore their potential role in pesticide resistance, specifically in response to CAP exposure. Genome sequencing helped identify *Mth* genes using model insects, such as *D. melanogaster*, *B. mori*, and *M. domestica*. This study expands our understanding of GPCR-mediated detoxification pathways in lepidopteran pests by demonstrating that specific *Mth* genes, particularly *PxMth2*, act as significant participants in resistance mechanisms. *Mth* genes are known for their roles in stress responses and longevity in insects [47,48]. Eight *Mth* genes were detected in the *P. xylostella* genome. The CAP-resistant strain of *P. xylostella* showed significant expression of these genes, suggesting that they might have a role in insects’ resistance to CAP exposure. This is in conformity with the results of other studies, underscoring the role of *Mth* genes in stress resistance [15,46]. Recent findings in *Zeugodacus cucurbitae* provide further support for the conserved role of GPCRs in insecticide resistance. A total of 80 GPCR genes were identified in *Z. cucurbitae*, including seven Methuselah-like GPCRs (*Mth*/*Mthl*). Expression profiling revealed that three Methuselah-like GPCR genes (MFZC63, MFZC66, MFZC67) showed high expression under β-cypermethrin stress [49], consistent with earlier reports linking *Mth*/*Mthl* genes to stress tolerance and survival in *Drosophila* [15,50].

RT-qPCR and genetic analyses were employed to investigate the expression of *Mth* genes in susceptible and resistant strains of *P. xylostella* under CAP exposure. The resistant strain exhibited significantly higher levels of *PxMth2* expression. Further analysis confirmed that both LC_30_ and LC_50_ doses of CAP upregulated *PxMth2*, highlighting a role in detoxification. These findings imply that *PxMth2* is a good candidate for further functional studies that aim to enhance pest management strategies.

The structure of *PxMth2*, containing seven TMHs, an intracellular domain, and an extracellular domain, aligns with the conserved characteristics of class B GPCRs. Notably, the presence of ten conserved cysteine residues in the ectodomain, along with hydrophobic and charged residues within the ligand-binding regions, underscores the structural and functional conservation of *Mth* receptors across species. These conserved elements, particularly secondary structural components (e.g., α-helices and β-sheets), emphasize the evolutionary stability of *Mth* frameworks. Structural integrity, confirmed by the Ramachandran plot, highlighted functional flexibility at key sites, correlating with evolutionary pressures, such as insecticide exposure. *PxMth2* likely interacts with CAP, mediating resistance through receptor sensitivity modulation. In the absence of CAP exposure, *Mth* genes—including *PxMth2*—play essential roles in regulating key physiological processes such as stress tolerance, aging, and innate immunity. The *methuselah* GPCR in *Drosophila* is best known for extending lifespan and bolstering resistance to oxidative damage, starvation, heat, and other stressors [47]. Similarly, *T. castaneum Mth*-like genes have been shown to influence development, stress resistance, lifespan, and reproduction [50]. Furthermore, *Mth* mutants demonstrate enhanced detoxification following exposure to chemicals, supporting roles in basal stress-response and homeostatic signaling [51]. Therefore, while *PxMth2* is strongly upregulated under CAP stress in our study, these findings suggest it also has inherent functions in maintaining homeostasis and environmental resilience under normal conditions.

RNAi was successfully used to silence *PxMth2* in third-instar larvae of *P. xylostella.* These findings conform to those of studies on *T. castaneum*, *D. helophoroides*, and *Lymantria dispar*, where silencing *Mth* like-genes reduced lifespan [18,38,45]. These findings indicate that *PxMth2* has a complex, critical role in regulating longevity in *P. xylostella*, implying that *PxMth2* contributes to insecticide resistance by transmitting extracellular signals to downstream genes in response to stress caused by insecticides [52]. The involvement of *PxMth2* in this stress response probably involves regulating the expression of downstream target genes (e.g., *GSTs* and *P450s*). Xenobiotic metabolism facilitated by GSTs or CYPs is widely recognized as a general mechanism for insecticide resistance [53,54,55]. A significant process that allows insects to adapt to plant secondary compounds and allelochemicals is the detoxification of xenobiotics by CYPs [56,57,58,59,60,61,62]. The expression of three examined *CYP6* genes (*CYP6B6, CYP6B7*, and *CYP6BF1*) was dramatically decreased in *P. xylostella* larvae when *PxMth2* expression was silenced, suggesting that *PxMth2* positively controls cytochrome P450 expression in this species. When *L. dispar* larvae were subjected to different pesticide stressors, several *P450* genes (e.g., *CYP6B53*, *CYP6AE51*, *CYP6CT4*, *CYP6AB36*, and *CYP6AN15v1*) showed distinct expression patterns [63]. Our results confirm that *PxMth2* could affect how *P450* genes are regulated in reaction to pesticide stress. Similarly, in *Culex pipiens pallens*, a GPCR-arrestin gene has been shown to regulate an insecticide-resistance-associated *CYP* gene in a deltamethrin-resistant strain [64]. Four GPCR-related genes were found to be important in controlling the expression of several resistance-related *P450* genes in *Culex quinquefasciatus* [65]. Furthermore, in mosquitoes, rhodopsin-like GPCRs play a role in insecticide resistance. When these receptors were knocked down, permethrin resistance decreased, along with the expression of resistance-related P450 and PKA genes. This role was further confirmed in transgenic *D. melanogaster*, and inhibition of cAMP or knockdown of PKA genes also reduced resistance and P450 expression [10]. These results demonstrate the unique function of GPCRs as key modulators of metabolic pesticide resistance mediated by *P450*.

According to our findings, *PxMth2* promotes stress resistance in *P. xylostella* by controlling genes linked to stress responses and detoxification, including *P450s*. These results advance our knowledge of a novel GPCR in insects and raise the possibility that it could be the target of insecticides that target GPCRs. Overall, our findings underscore the critical role of *Mths*, particularly those involved in stress responses and detoxification, in *P. xylostella* resistance to CAP. This research provides a foundation for developing innovative pest control strategies that are more target specific and minimize environmental impacts. However, further field studies are necessary to confirm our findings and fully explore GPCR-mediated resistance mechanisms in *P. xylostella*.

## 5. Conclusions

In this study, we identified and characterized *Mth* genes in *P. xylostella*, revealing that *PxMth2* plays a role in pesticide resistance, particularly against CAP. *PxMth2* was significantly upregulated in resistant strains and under CAP exposure, and RNAi demonstrated its involvement in regulating downstream detoxification genes, including (*CYP6B6*, *CYP6B7*, *CYP6BF1*). Structural and evolutionary analyses confirmed the conservation of *PxMth2* as a class B GPCR, supporting its functional significance in stress response and homeostasis. These findings highlight *PxMth2* as a target for future pest management strategies and contribute to our understanding of GPCR-mediated detoxification pathways in lepidopteran pests.

## Figures and Tables

**Table 1:** Identified candidate *Mth* genes in *P. xylostella* based on a comparison with known *Mth* genes in *Bombyx mori*, *D. melanogaster*, and *Musca domestica*. Details come from publicly available databases (see text for details).

Gene ID	Rename	Chr	Start	End	Strand	A.A	Exon	Intron	MW (kDs)	PI	GRAVY
LOC105382322	*PXMTH1*	25	4,719,631	4,726,549	F	493	9	8	56.67	6.23	0.326
LOC119693055	*PXMTH2*	8	3,392,336	3,414,747	R	514	10	9	57.1	8.05	0.215
LOC105397179	*PXMTH3*	25	4,705,051	4,71,5964	F	407	9	8	45.86	7.81	0.434
LOC105392921	*PXMTH4*	4	7,187,785	7,198,855	F	715	9	8	79.82	7.65	0.002
LOC105382325	*PXMTH5*	25	4,680,720	4,685,582	F	423	8	7	47.14	8.13	0.365
LOC105383750	*PXMTH6*	8	2,418,343	2,433,086	F	565	9	8	64.67	7.99	0.081
LOC119693713	*PXMTH7*	10	148,466	152,815	F	583	2	1	59.84	8.76	0.408
LOC105380334	*PXMTH8*	8	8,697,740	8,754,344	R	666	10	9	74.42	8.39	−0.019

**Table 2:** Toxicity bioassay of chlorantraniliprole on *P. xylostella*.

Insecticide	Population	N ^a^	LC_50_ (mg/L)	95% CI of LC_50_^b^	X^2^ (df) ^c^	RR ^d^
Chlorantraniliprole	Resistant	150	63.2	46.66-70.2	0.90 (3)	42.1
	Control	150	1.5	0.8-2.3	0.90 (3)	1

^a^ The overall number of insects used at all concentrations. ^b^ A 95% confidence interval. ^c^ Chi-square test with those in brackets demonstrating the degree of freedom. ^d^ RR: Resistance ratio = LC_50_ of the susceptible population/LC_50_ of the resistant population.

**Table 3:** Toxicity bioassay of chlorantraniliprole on Transgenic and Wild-Type *Drosophila melanogaster*.

Insecticide	Population	N ^a^	LC_50_ (mg/L)	95% CI of LC_50_^b^	X^2^ (df) ^c^	RR ^d^
chlorantraniliprole	Transgenic *Drosophila*	420	0.7	0.6–1	7 (4)	2.9
	Control (WT)	420	0.24	0.21–0.28	1.57 (4)	1

^a^ Total number of insects tested across all concentrations. ^b^ Values represent the 95% confidence interval. ^c^ Chi-square test results, with degrees of freedom indicated in parentheses. ^d^ RR (Resistance Ratio) calculated as LC_50_ of the susceptible/control strain divided by the LC_50_ of the transgenic *Drosophila* strain.

## Data Availability

The original contributions presented in this study are included in the article/Supplementary Material. Further inquiries can be directed to the corresponding author.

## Supplementary Materials

The following supporting information can be downloaded at: https://www.mdpi.com/article/10.3390/insects16111092/s1, Table S1: List of primers used for qPCR experiment.

## References

[1] Garland S.L. (2013). Are GPCRs still a source of new targets?. J. Biomol. Screen..

[2] Filmore D. (2004). It’s a GPCR world. Mod. Drug Discov..

[3] Klabunde T., Hessler G. (2002). Drug design strategies for targeting G-protein-coupled receptors. Chembiochem.

[4] Liu N., Li T., Wang Y., Liu S. (2021). G-protein coupled receptors (GPCRs) in insects—A potential target for new insecticide development. Molecules.

[5] Syrovatkina V., Alegre K.O., Dey R., Huang X.-Y. (2016). Regulation, signaling, and physiological functions of G-proteins. J. Mol. Biol..

[6] Falkenburger B.H., Dickson E.J., Hille B. (2013). Quantitative properties and receptor reserve of the DAG and PKC branch of Gq-coupled receptor signaling. J. Gen. Physiol..

[7] Kim C., Cheng C.Y., Saldanha S.A., Taylor S.S. (2007). PKA-I holoenzyme structure reveals a mechanism for cAMP-dependent activation. Cell.

[8] Chen-Goodspeed M., Lukan A.N., Dessauer C.W. (2005). Modeling of Gαs and Gαi regulation of human type V and VI adenylyl cyclase. J. Biol. Chem..

[9] Bradley J., Li J., Davidson N., Lester H.A., Zinn K. (1994). Heteromeric olfactory cyclic nucleotide-gated channels: A subunit that confers increased sensitivity to cAMP. Proc. Natl. Acad. Sci. USA.

[10] Li T., Cao C., Yang T., Zhang L., He L., Xi Z., Bian G., Liu N. (2015). A G-protein-coupled receptor regulation pathway in cytochrome P450-mediated permethrin-resistance in mosquitoes, *Culex quinquefasciatus*. Sci. Rep..

[11] Li T., Liu N. (2018). The function of G-protein-coupled receptor-regulatory cascade in southern house mosquitoes (Diptera: Culicidae). J. Med. Entomol..

[12] Audsley N., Down R.E. (2015). G protein coupled receptors as targets for next generation pesticides. Insect Biochem. Mol. Biol..

[13] Patel M.V., Hallal D.A., Jones J.W., Bronner D.N., Zein R., Caravas J., Husain Z., Friedrich M., Vanberkum M.F. (2012). Dramatic expansion and developmental expression diversification of the methuselah gene family during recent *Drosophila* evolution. J. Exp. Zool. B Mol. Dev. Evol..

[14] Song W., Ranjan R., Dawson-Scully K., Bronk P., Marin L., Seroude L., Lin Y.J., Nie Z., Atwood H.L., Benzer S. (2002). Presynaptic regulation of neu rotransmission in *Drosophila* by the g protein-coupled receptor methuselah. Neuron.

[15] Lin Y.-J., Seroude L., Benzer S. (1998). Extended life-span and stress resistance in the *Drosophila* mutant methuselah. Science.

[16] Wang J., Wang Z., Zhang Z., Hua Q., Wang M., Shi C., Xue L., Zhang R., Xie X. (2015). Methuselah regulates longevity via dTOR: A pathway revealed by small-molecule ligands. J. Mol. Cell Biol..

[17] Li C., Wu W., Sang M., Liu X., Hu X., Yun X., Li B. (2014). Comparative RNA-sequencing analysis of *mthl1* functions and signal transductions in *Tribolium castaneum*. Gene.

[18] Zhang Z.Y., Zhang J., Yang C.J., Lian H.Y., Yu H., Huang X.M., Cai P. (2016). Coupling mechanism of electromagnetic field and thermal stress on *Drosophila melanogaster*. PLoS ONE.

[19] Furlong M.J., Wright D.J., Dosdall L.M. (2013). Diamondback moth ecology and management: Problems, progress, and prospects. Annu. Rev. Entomol..

[20] Tabashnik B.E., Huang F., Ghimire M.N., Leonard B.R., Siegfried B.D., Rangasamy M., Yang Y., Wu Y., Gahan L.J., Heckel D.G. (2011). Efficacy of genetically modified Bt toxins against insects with different genetic mechanisms of resistance. Nat. Biotechnol..

[21] You M., Yue Z., He W., Yang X., Yang G., Xie M., Zhan D., Baxter S.W., Vasseur L., Gurr G.M. (2013). A heterozygous moth genome provides insights into herbivory and detoxification. Nat. Genet..

[22] Xu P., Lu B., Xiao H., Fu X., Murphy R.W., Wu K. (2013). The evolution and expression of the moth visual opsin family. PLoS ONE.

[23] Lee D.W., Shrestha S., Kim A.Y., Park S.J., Yang C.Y., Kim Y., Koh Y.H. (2011). RNA interference of pheromone biosynthesis activating neuropeptide receptor suppresses mating behavior by inhibiting sex pheromone production in *Plutella xylostella* (L.). Insect Biochem. Mol. Biol..

[24] Yin F., Lin Q., Wang X., Li Z., Feng X., Shabbir M.Z. (2021). The glutathione S-transferase (PxGST2L) may contribute to the detoxification metabolism of chlorantraniliprole in *Plutella xylostella* (L.). Ecotoxicology.

[25] Zolfaghari M., Yin F., Jurat-Fuentes J.L., Xiao Y., Peng Z., Wang J., Yang X., Li Z.-Y. (2024). Effects of *Bacillus thuringiensis* Treatment on Expression of Detoxification Genes in Chlorantraniliprole-Resistant *Plutella xylostella*. Insects.

[26] Zolfaghari M., Ghadamyari M., Sajedi R.H. (2019). Resistance Mechanisms of a Field Population of Diamondback Moth, *Plutella xylostella* (Lepidoptera: Plutellidae), to Current Organophosphate Pesticides. J. Crop Prot..

[27] Tabashnik T., Cushing N. (1987). Leaf residue vs. topical bioassays for assessing insecticide resistance in the diamond-back moth, *Plutella xylostella* L. FAO Plant Prot. Bull..

[28] Kahsay R.Y., Gao G., Liao L. (2005). An improved hidden Markov model for transmembrane protein detection and topology prediction and its applications to complete genomes. Bioinformatics.

[29] Horton P., Park K.-J., Obayashi T., Fujita N., Harada H., Adams-Collier C., Nakai K. (2007). WoLF PSORT: Protein localization predictor. Nucleic Acids Res..

[30] Tamura K., Peterson D., Peterson N., Stecher G., Nei M., Kumar S. (2011). MEGA5: Molecular evolutionary genetics analysis using maximum likelihood, evolutionary distance, and maximum parsimony methods. Mol. Biol. Evol..

[31] Chen C., Chen H., Zhang Y., Thomas H.R., Frank M.H., He Y., Xia R. (2020). TBtools: An integrative toolkit developed for interactive analyses of big biological data. Mol. Plant.

[32] Chao J., Li Z., Sun Y., Aluko O.O., Wu X., Wang Q., Liu G. (2021). MG2C: A user-friendly online tool for drawing genetic maps. Mol. Hortic..

[33] Guo A.-Y., Zhu Q.-H., Chen X., Luo J.-C. (2007). GSDS: A gene structure display server. Yi Chuan Hered..

[34] Bailey T.L., Williams N., Misleh C., Li W.W. (2006). MEME: Discovering and analyzing DNA and protein sequence motifs. Nucleic Acids Res..

[35] Teufel F., Almagro Armenteros J.J., Johansen A.R., Gíslason M.H., Pihl S.I., Tsirigos K.D., Winther O., Brunak S., Von Heijne G., Nielsen H. (2022). SignalP 6.0 predicts all five types of signal peptides using protein language models. Nat. Biotechnol..

[36] Duckert P., Brunak S., Blom N. (2004). Prediction of proprotein convertase cleavage sites. Protein Eng. Des. Sel..

[37] Rinkevich F.D., Scott J.G. (2012). Reduction of dADAR activity affects the sensitivity of *Drosophila melanogaster* to spinosad and imidacloprid. Pestic. Biochem. Physiol..

[38] Cao C., Sun L., Du H., Moural T.W., Bai H., Liu P., Zhu F. (2019). Physiological functions of a methuselah-like G protein coupled receptor in *Lymantria dispar* Linnaeus. Pestic. Biochem. Physiol..

[39] Sun L., Liu P., Zhang C., Du H., Wang Z., Moural T.W., Zhu F., Cao C. (2019). Ocular albinism type 1 (OA1) regulates deltamethrin tolerance in *Lymantria dispar* and *Drosophila melanogaster*. Front. Physiol..

[40] Yu Y., Zhou P., Zhang J., Zheng C., Zhang J., Chen N. (2018). Pheromone-binding proteins in the Asian gypsy moth females, *Lymantria dispar*, recognizing the sex pheromone and plant volatiles. Arch. Insect Biochem. Physiol..

[41] Xu J., Wang X.F., Chen P., Liu F.T., Zheng S.C., Ye H., Mo M.H. (2016). RNA interference in moths: Mechanisms, applications, and progress. Genes.

[42] Kim Y.H., Issa M.S., Cooper A.M., Zhu K.Y. (2015). RNA interference: Applications and advances in insect toxicology and insect pest management. Pestic. Biochem. Physiol..

[43] Lin W., Yu Y., Zhou P., Zhang J., Dou L., Hao Q., Chen H., Zhu S. (2015). Identification and knockdown of the olfactory receptor (OrCo) in gypsy moth, *Lymantria dispar*. Int. J. Biol. Sci..

[44] Terenius O., Papanicolaou A., Garbutt J.S., Eleftherianos I., Huvenne H., Kanginakudru S., Albrechtsen M., An C., Aymeric J.L., Barthel A. (2011). RNA interference in Lepidoptera: An overview of successful and unsuccessful studies and implications for experimental design. J. Insect Physiol..

[45] Li C., Zhang Y., Yun X., Wang Y., Sang M., Liu X., Hu X., Li B. (2014). Methuselah-like genes affect development, stress resistance, lifespan and reproduction in *Tribolium castaneum*. Insect Mol. Biol..

[46] Gimenez L.E., Ghildyal P., Fischer K.E., Hu H., Ja W.W., Eaton B.A., Wu Y., Austad S.N., Ranjan R. (2013). Modulation of methuselah expression targeted to *Drosophila* insulin-producing cells extends life and enhances oxidative stress resistance. Aging Cell.

[47] Friedrich M., Jones J.W. (2016). Gene ages, nomenclatures, and functional diversification of the Methuselah/Methuselah—Like GPCR family in *Drosophila* and *Tribolium*. J. Exp. Zool. Part B.

[48] Li C., Chen M., Sang M., Liu X., Wu W., Li B. (2013). Comparative genomic analysis and evolution of family-B G protein-coupled receptors from six model insect species. Gene.

[49] Shabbir S., Deng M.-G., Nawaz M., Lin Q.-S. (2024). Genome-Wide Identification of G-Protein Coupled Receptors (GPCRs) and Their Expression Profile in Response to β-Cypermethrin Stress in *Zeugodacus cucurbitae*. Pestic. Biochem. Physiol..

[50] Li C., Song X., Chen X., Liu X., Sang M., Wu W., Yun X., Hu X., Li B. (2014). Identification and comparative analysis of G protein-coupled receptors in *Pediculus humanus humanus*. Genomics.

[51] Pandey A., Khatoon R., Saini S., Vimal D., Patel D.K., Narayan G., Chowdhuri D.K. (2015). Efficacy of methuselah gene mutation toward tolerance of dichlorvos exposure in *Drosophila melanogaster*. Free Radic. Biol. Med..

[52] Rosenbaum D.M., Rasmussen S.G., Kobilka B.K. (2009). The structure and function of G-protein-coupled receptors. Nature.

[53] Despres L., David J.P., Gallet C. (2007). The evolutionary ecology of insect resistance to plant chemicals. Trends Ecol. Evol..

[54] Liu N., Zhu F., Xu Q., Pridgeon J.W., Gao X. (2006). Behavioral change, physiological modification, and metabolic detoxification: Mechanisms of insecticide resistance. Acta Entomol. Sin..

[55] Ranson H., Claudianos C., Ortelli F., Abgrall C., Hemingway J., Sharakhova M.V., Unger M.F., Collins F.H., Feyereisen R. (2002). Evolution of supergene families associated with insecticide resistance. Science.

[56] Zhu F., Gujar H., Gordon J.R., Haynes K.F., Potter M.F., Palli S.R. (2013). Bed bugs evolved unique adaptive strategy to resist pyrethroid insecticides. Sci. Rep..

[57] Zhu F., Moural T.W., Shah K., Palli S.R. (2013). Integrated analysis of cytochrome P450 gene superfamily in the red flour beetle, *Tribolium castaneum*. BMC Genom..

[58] Feyereisen R., Gilbert L.I. (2012). Insect CYP genes and P450 enzymes. Insect Molecular Biology and Biochemistry.

[59] Liu N., Zhu F., Liu T., Kang L. (2011). House fly cytochrome P450s: Their role in insecticide resistance and strategies in the isolation and characterization. Recent Advances in Entomological Research.

[60] Zhu F., Parthasarathy R., Bai H., Woithe K., Kaussmann M., Nauen R., Harrison D.A., Palli S.R. (2010). A brain-specific cytochrome P450 responsible for the majority of deltamethrin resistance in the QTC279 strain of *Tribolium castaneum*. Proc. Natl. Acad. Sci. USA.

[61] Zhu F., Liu N. (2008). Differential expression of *CYP6A5* and *CYP6A5v2* in pyrethroid resistant house flies, *Musca domestica*. Arch. Insect Biochem. Physiol..

[62] Scott J.G., Liu N., Wen Z. (1998). Insect cytochromes P450: Diversity, insecticide resistance and tolerance to plant toxins. Comp. Biochem. Physiol. Part C Pharmacol. Toxicol. Endocrinol..

[63] Sun L., Wang Z., Zou C., Cao C. (2014). Transcription profiling of 12 Asian gypsy moth (*Lymantria dispar*) cytochrome P450 genes in response to insecticides. Arch. Insect Biochem. Physiol..

[64] Sun Y., Zou P., Yu X.Y., Chen C., Yu J., Shi L.N., Hong S.C., Zhou D., Chang X.L., Wang W.J. (2012). Functional characterization of an arrestin gene on insecticide resistance of *Culex pipiens pallens*. Parasites Vectors.

[65] Li T., Liu L., Zhang L., Liu N. (2014). Role of G-protein-coupled receptor-related genes in insecticide resistance of the mosquito, *Culex quinquefasciatus*. Sci. Rep..

